# Challenges in implementing yearly enhanced safety surveillance of influenza vaccination in Europe: lessons learned and future perspectives

**DOI:** 10.1080/21645515.2019.1608745

**Published:** 2019-05-22

**Authors:** Gaël Dos Santos

**Affiliations:** US/BE Vaccine Research and Development Center, Clinical R&D, GSK, Wavre, Belgium

**Keywords:** Pharmacovigilance, enhanced safety surveillance, adverse events following immunization, European Medicines Agency, influenza vaccine, safety signal

## Abstract

Seasonal influenza vaccines are frequently reformulated, leading to specific challenges for continuous benefit/risk monitoring. In 2014, the European Medicines Agency started requiring annual enhanced safety surveillance (ESS). This article provides a perspective on ESS studies conducted ever since and aims to map existing initiatives used to monitor adverse events following influenza immunization. Of 11 ESS studies, reporting surveillance data of at least five different vaccine brands during four seasons, all were able to rapidly capture vaccine-specific adverse events of interest reports. However, challenges have been identified during study implementation, including recruitment of sufficient participants, enrolling younger age groups, collecting data of vaccine batch numbers, comparing observed with expected rates and achieving adequate return of reported events. Harmonizing safety monitoring standards across countries, and bridging between routine pharmacovigilance and ESS, is likely to allow more comprehensive assessments of influenza vaccine safety, requiring close collaboration between regulators, public health, and manufacturers.

## Introduction

Immunization remains the cornerstone of influenza prevention. Due to frequent genetic and antigenic changes in influenza viruses, seasonal influenza vaccines require frequently reformulation. Based on surveillance data, the World Health Organization (WHO) issues twice-yearly recommendations for influenza vaccine composition for the northern and southern hemisphere seasons, with these used by vaccine manufacturers and regulatory authorities to develop and license seasonal vaccines.^1^

Across the European Union (EU) annual influenza vaccination is recommended in older adults (≥60 or ≥65 years), with most countries also recommending immunization in pregnancy, and some, recommending pediatric vaccination.^2^ As described by Vaccines Europe influenza working group, uptake is however suboptimal, with a study conducted in 2014 in 27 EU countries reporting that only approximately 80 million out of 180 million eligible persons (44%) were vaccinated.^3^ Most recent estimates of annual coverage in older adults in Europe range from 4.3% in Poland to 71.6% in the UK.^2^ Initiatives to improve influenza vaccine coverage is needed, especially given the substantial health benefits; a modeling study estimated that across the EU, seasonal influenza vaccination would prevent an average of 1.6 million to 2.1 million influenza cases each year, and 45,300–65,600 hospitalizations, with 25,200–37,200 fewer deaths.^3^

Pharmacovigilance studies are an important aspect of monitoring influenza vaccine safety and can help maintain public and professional confidence which in turn may improve vaccine uptake. In the EU, such studies are performed under guidance from the European Medicines Agency (EMA), although changes to specific requirements for influenza vaccine pharmacovigilance have been made in recent years.^4,5^

The purpose of this article is to present an explanation of these changes and an overview of enhanced safety surveillance (ESS) studies performed by vaccine manufacturers in Europe since the introduction of the new regulatory framework. Surveillance systems used out with Europe and some initiatives to gather safety data are also discussed.

To facilitate this review, a literature search was conducted, screening PubMed, Cochrane library, and Embase databases for English language publications from January 2014 to January 2019 using search terms “influenza/flu vaccination” or “vaccine” and “ safety surveillance” in combination. Search results were limited to reports from Europe. The EU post-authorization safety study (PASS) register database and gray literature were also searched in a similar manner.

## Changes to EMA guidance

Until recently, the EMA required manufacturers of seasonal influenza vaccines to conduct small-scale safety and immunogenicity clinical trials in two groups of at least 50 subjects aged 18−60 years and >60 years.^6^ This requirement was withdrawn in 2015,^7^ on the basis that: (1) such studies were not sufficiently informative (or adequately powered) to detect seasonal vaccine efficacy and safety; (2) the substantive body of existing safety data reported in surveillance studies; and (3) the recognition that annual antigenic drift and corresponding changes in vaccine strains are unlikely to substantively affect vaccine safety. However, given that seasonal influenza vaccines are likely to be reformulated annually, the need for near real-time benefit-risk monitoring is acknowledged.^7^ The EMA guidance required such trials to be replaced by ESS studies as part of post-marketing monitoring programs, as well as vaccine effectiveness assessments.^7^

To that end, the EMA released interim guidance in 2014,^4^ and provided complementary information in 2016 in the Guideline on Influenza Vaccines,^5^ requesting all manufacturers to conduct annual ESS, with the objective of rapidly detecting and evaluating potential new safety concerns. Each manufacturer is required to specify in their Risk Management Plan (RMP) their proposed ESS approach. A requirement is that safety and reactogenicity of each new formulation are evaluated in terms of local and systemic adverse reactions in the age groups in which the vaccine is to be used, with particular attention paid to young children, if applicable. These aspects may not necessarily apply at a national level – where submissions for nationally authorized products do not necessarily require an RMP or annual ESS.

The ultimate purpose is to identify and ideally mitigate risks early in the season (i.e. at least within the first month after the start of the seasonal immunization period), with results provided in a timely manner to competent regulatory authorities. Any clinically significant changes in the severity or frequency of reactogenicity from the previous vaccine composition may indicate a potential safety concern that deserves further investigation. For example, an increase in severity or frequency of fever could indicate a potentially higher risk of febrile convulsion; prompt identification of safety signals is expected to permit earlier risk mitigation. This was the case during the 2010 influenza season in Australia, where surveillance detected increased convulsion rates in young children receiving Fluvax (CSL Biotherapies).^8^ Despite subsequent vaccine deregistration for children <5 years, continued public concerns about vaccine safety may have contributed to significantly lower uptake of influenza vaccine during subsequent seasons.^9^ A similar example occurred more recently in Italy when, following three deaths in older adults, the Italian Medicines Agency suspended the use of two batches of Fluad (Seqirus) as a precautionary measure on 27 November 2014, leading to multiple investigations, although the EMA ultimately concluded that there was no evidence of a causal association.^10^

Expansion of national vaccination programs in Europe to include new target groups such as healthy children and pregnant women creates additional demand for information and reassurance on the balance of risks and benefits. However, several particular challenges are associated with routine pharmacovigilance of seasonal influenza vaccines. For example, as mass immunization occurs during a relatively short period each year, with close temporality between virus circulation and launch of the annual vaccination campaign, detection of safety issues in ‘real time’ is challenging if one relies solely on routine pharmacovigilance; it cannot be ruled out that some safety concerns could be detected only by the end of the influenza season. Furthermore, EU market diversity for seasonal influenza vaccines poses additional challenges for routine pharmacovigilance (in terms of both the variety of available vaccines and also the variety of authorization routes, national immunization policies, and operational infrastructures for vaccine administration). Pragmatically, as vaccine allocation and distribution in the EU market is essentially based on annual tenders, it is a considerable challenge to identify suitable regions/countries well in advance of the upcoming influenza season to conduct the ESS, and to replicate the initiative in the same participating countries every year.

## Influenza ESS conducted in the EU since the EMA guidance release

Current EMA guidance includes a list of pre-defined adverse events of interest (AEIs) to be monitored during safety surveillance, and suggests three monitoring options for vaccine manufacturers: (1) active surveillance, using existing methods of post-authorization surveillance; (2) enhanced passive surveillance (EPS) in which vaccine use is estimated early in the season and additional steps are taken to facilitate passive reporting of AEIs; and (3) data mining or other use of electronic health records (EHRs).^4,11,12^

Four complete influenza seasons have elapsed since the release of the EMA interim and updated guidance on ESS and several groups (i.e., academic groups, public health representatives, and manufacturers) have reported the outcome of their ESS investigations for the following seasons; 2014/15 (two studies), 2015/16 (three studies), 2016/17 (three studies); and 2017/18 (three studies) (Table 1). Most studies are published^13-20^ (or accepted for publication^21^), and one additional study evaluating GSKs Fluarix Tetra for the 2017/18 (NCT03278067) season is also completed.^22^

**Table 1.** ESS implemented in EU since the release of EMA guidance on safety surveillance for seasonal influenza vaccines.

						Most frequently reported AE^b^		
Study ID or Study Reference	Country	Study approach and data collection method	Vaccine brand and manufacturer	Number of participants (age group)	Safety outcomes – any AE^a^	AE	Frequency	Conclusions reported by the authors
**2014–2015**								
Demeulemeester et al.^13^	Belgium	EPS; safety diary card and face-to-face interview	Intanza (9 µg and 15 µg),Sanofi Pasteur	Intanza 9 µg:105 (18−59 years); Intanza 15 µg:105 (≥60 years)	Intanza 9 µg AE:81.9%	Injection site pain	Very common	No safety signal for influenza vaccines were identified. EPS using data from safety diaries is feasible and timely
						Injection site erythema*	Very common	
						Injection site pruritus	Very common	
						Myalgia	Very common	
						Headache	Very common	
					Intanza 15 µg AE: 62.9%	Injection site erythema	Very common	
						Injection site pruritus	Very common	
						Injection site pain	Very common	
						Headache	Very common	
						Myalgia	Very common	
McNaughton et al.^14^	England,UK	Non-interventional cohort PASS;web-based and/or paper questionnaire	Fluenz Tetra (QLAIV), AstraZeneca	385 (2–17 years)	AE: 61.6%	Nasal congestion/runny nose	Very common	No safety signal for influenza vaccines were identified. Use of web-based and/or paper questionnaire can generate rapid safety data
						Malaise	Very common	
						Cough	Very common	
						Decreased appetite	Very common	
						Increased irritability (if the child is between 2 and 4 years)	Very common	
**2015–2016**								
De Lusignan et al., accepted^21^	England,UK	EPS; AERC (Fluarix Tetra only^c^) and EHR	Fluarix Tetra, GSK^d^;Other vaccine brands	Fluarix Tetra: 3616 (≥6 months);Other brands: 12,247 (≥6 months)	Fluarix Tetra: AE: 2.6%	Muscles aches/Myalgia	Uncommon	No safety signal for influenza vaccines were identified. Data mining from EHR record data supplemented by AERC data is a feasible method for EPS.
						Cough	Uncommon	
						Fever with temperature	Uncommon	
						Nasal congestion	Uncommon	
						Fatigue	Uncommon	
						Fever	Uncommon	
						Oropharyngeal pain	Uncommon	
					Other brands: AE: 3.0%	Fever with temperature	Uncommon	
						Cough	Uncommon	
						Muscles aches/Myalgia	Uncommon	
						Headache	Uncommon	
						generalized rash	Uncommon	
Bricout et al.^15^	UK and Finland	EPSAERC and EHR	Vaxigrip and Intanza 15 µg, Sanofi Pasteur	Vaxigrip: 1012 (≥6 months) Intanza: 1017 (≥60 years; UK only)	Vaxigrip;AE: 3.2%	Local pain	Common	No safety signal for influenza vaccines were identified. Data mining from EHR record data supplemented by AERC data is a feasible method for EPS.
						Cough	Common	
						Pyrexia	Common	
						Rhinorrhea	Common	
						Influenza-like illness	Common	
						Malaise	Common	
					Intanza: AE: 3.0%	Local pain	Common	
Spila Alegiani et al.^16^	Italy	EPS;telephone interview (or web-based questionnaire)	Various vaccines (brand not stated^e^), Subunit vaccine Split vaccine, Adjuvanted vaccine, Intradermal	≥6 months Subunit vaccine: 1636 Split vaccine: 660 Adjuvanted vaccine: 526 Intradermal: 394	Subunit vaccine: AE: 27.1%	Local pain	Very common	
						Local swelling	Common	
						Local Induration	Common	
						Local redness	Common	
						Malaise	Common	
					Split vaccine AE: 20.9%	Local pain	Very common	
						Local swelling	Common	
						Local redness	Common	
						Local Induration	Common	
						Malaise	Common	
						Myalgia	Common	
					Adjuvanted vaccine AE: 21.7%	Local pain	Very common	
						Malaise	Common	
						Local swelling	Common	
						Local Induration	Common	
						Local redness	Common	
					Intradermal AE: 38.1%	Local redness	Very common	
						Local swelling	Very common	
						Local Induration	Very common	
						Local pain	Very common	
						Malaise	Common	
**2016–2017**								
Chabanon et al.^17^	UK and Republic of Ireland	EPS;AERC and EHR	Vaxigrip and Intanza 15 µg, Sanofi Pasteur	Vaxigrip: 962 (≥6 months) Intanza: 1000 (≥60 years; K only)	Vaxigrip;AE: 1.8%	Pyrexia	Uncommon	No safety signal for influenza vaccines were identified. Conducting EPS in successive years can contribute to sustainable safety surveillance earlier in the season.
						Headache	Uncommon	
						Malaise	Uncommon	
						Local erythema	Uncommon	
					Intanza: AE: 2.1%	Oropharyngeal pain	Uncommon	
						Headache	Uncommon	
						Malaise	Uncommon	
						Local erythema	Uncommon	
						Cough	Uncommon	
						Rhinorrhea	Uncommon	
						Local swelling	Uncommon	
Stuurman et al.^18^	Belgium	EPS;web-based questionnaire	Alpharix Tetra, GSK^d^	100 (18–65 years)	AE: 65% (general reactions) -68% (local reactions)	Local pain	Very common	No safety signal for influenza vaccines were identified Use of web-based questionnaire to collect safety data early in the season is feasible.
						Headache	Very common	
						Local Induration	Very common	
						Myalgia/arthralgia	Very common	
						Malaise	Very common	
De Lusignan et al.^19^	England,UK	EPS;AERC (Fluarix Tetra only^c^) and EHR	Fluarix Tetra, GSK^d^ Other vaccine, brands Unknown vaccine	Fluarix Tetra: 13,861 (≥6 months) Other brands: 2295 (≥6 months) Unknown vaccine: 3178 (≥6 months)	Fluarix Tetra: AE: 7.6%	Muscle aches/myalgia	Common	No safety signal for influenza vaccines were identified. Combining routine EHR record data with AERC data reporting may improve capture of AEIs compared with classical passive surveillance alone.
						Nasal congestion	Common	
						Rhinorrhea	Common	
						Cough	Common	
						Headache	Common	
					Other brands: AE: 2.6%	Fever/pyrexia	Common	
						Cough	Uncommon	
						Muscle aches/myalgia	Uncommon	
						generalised rash	Uncommon	
						Headache	Uncommon	
					Unknown vaccine: AE: 1.8%	Fever/pyrexia	Uncommon	
						Muscle aches/myalgia	Uncommon	
						Cough	Uncommon	
						Headache	Uncommon	
						Diarrhea	Rare	
						generalised rash	Rare	
**2017–2018**								
NCT03278067^22^	England,UK	EPS;AERC (Fluarix Tetra only^d^) and EHR	Fluarix Tetra, GSK^e^ Other vaccine, brands Unknown vaccine	Fluarix Tetra: 16,433 (≥6 months) Other brands: 3310 (≥6 months) Unknown vaccine: 4196 (≥6 months)	Fluarix Tetra: AE: 9.2%	Muscle aches/myalgia	Common	No safety signal for influenza vaccines were identified. Combining routine EHR record data with AERC data reporting may improve capture of AEIs compared with classical passive surveillance alone.
						Rhinorrhea	Common	
						Cough	Common	
						Headache	Common	
						Local erythema	Common	
					Other brands: AE: 4.0%	Cough	Common	
						Rhinorrhea	Uncommon	
						Headache	Uncommon	
						Muscle aches/myalgia	Uncommon	
						Local erythema	Uncommon	
					Unknown vaccine: AE: 1.6%	Cough	Uncommon	
						Muscle aches/myalgia	Uncommon	
						Oropharyngeal pain	Uncommon	
						Headache	Uncommon	
						Conjunctivitis	Uncommon	
Alguacil-Ramos et al.^23^	Spain	Passive surveillance using a population-based description study design	Subunit vaccine, Adjuvanted vaccine (Brands not informed)	Subunit vaccine: 540,530 doses administered (≥6 months) Adjuvanted vaccine: 169,588 doses administered (≥18 years)	Subunit vaccine: AE: 0.70/10,000	Injection site reactions	Very rare	No safety signal for influenza vaccines were identified.
						Allergic/hypersensitivity reactions	Very rare	
						Fever	Very rare	
						Rash	Very rare	
						Headache	Very rare	
					Adjuvanted vaccine: AE: 1.00/10,000	Injection site reactions	Very rare	
						Fever	Very rare	
						Headache	Very rare	
						Decrease appetite	Very rare	
						Rash	Very rare	
						Myalgia	Very rare	
						Arthralgia	Very rare	
						Allergic/hypersensitivity reactions	Very rare	
Gandhi-Banga et al.^20^	UK and Republic of Ireland	EPS;safety report cards distributed to participants;telephone interview	Intanza 15 µg, Vaxigrip and Vaxigrip Tetra, Sanofi Pasteur	Intanza 15 µg: 979 (≥60 years) Vaxigrip: 1005 (≥6 months) Vaxigrip Tetra: 957 (≥3 years)	Intanza 15 µg: AE: 2.9%	Vaccination site inflammation	Common	No safety signal compared to other seasons for Intanza 15 µg and Vaxigrip. First season of licensure of Vaxigrip Tetra: AR frequencies similar to thosein the SmPC
						Vaccination site pruritus	Uncommon	
						Vaccination site erythema	Uncommon	
						Vaccination site reaction	Uncommon	
						Oropharyngeal pain	Uncommon	
						Hyperhidrosis	Uncommon	
					Vaxigrip: AE: 1.4%	Vaccination site inflammation	Uncommon	
						Headache	Uncommon	
						Malaise	Uncommon	
						Nausea	Uncommon	
						Lethargy	Uncommon	
						Oropharyngeal pain	Uncommon	
						Hyperhidrosis	Uncommon	
					Vaxigrip Tetra: AE: 2.1%	Headache	Uncommon	
						Fever	Uncommon	
						Vaccination site inflammation	Uncommon	
						Fatigue	Uncommon	
						Oropharyngeal pain	Uncommon	

^a^ Safety outcomes are reported as the % of subjects reporting an adverse event

^b^ Five most frequent AEs reported (or more if AEs are reported at equal rates). Frequencies are defined as: Very common: ≥1/10; Common ≥1/100 to <1/10; Uncommon: ≥1/1000 to <1/100; Rare: ≥1/10,000 to <1/1000; Very rare: <1/10,000

^c^ Data from the EHR for other vaccine brands used by the practices participating in the study were also collected

^d^ Fluarix Tetra and Alpharix Tetra are different brand names for the same vaccine

^e^ Non-manufacturer study sponsored by the Italian Medicines Agency (AIFA) AERC: adverse event reporting card; EHR: electronic health record; EMA: European Medicines Agency; EPS: enhanced passive surveillance; ESS: enhanced safety surveillance; EU: European Union; NA: not applicable; PASS: post-authorization safety study; QLAIV: quadrivalent live attenuated influenza vaccine; UK: United Kingdom

The vaccines studied were Sanofi Pasteur‘s Intanza (9 and 15 µg), Vaxigrip and Vaxigrip Tetra, GSK‘s Fluarix Tetra/Alpharix Tetra and AstraZeneca‘s Fluenz Tetra. The enrolled population varied from 100 to 710,118 vaccinees. Most studies evaluated safety in the UK (seven studies), with two conducted in Belgium, two in the Republic of Ireland, with data from recipients from Finland also reported. Two non-industry sponsored studies have also been conducted in Italy and Spain.

Of 11 studies reported here, 9 employed the EPS methodology,1 was set up as a non-interventional voluntary PASS, and 1 was a passive surveillance using a population-based description study design (Table 1). To facilitate passive reporting of AEIs, five of the EPS studies used a combination of Adverse Event Reporting Cards (AERCs) and data from patients‘ EHR,;^15,17,19,21,22^ one study used daily diaries and patient interviews,^13^ one used a web-based questionnaire with daily reminders via text message,^18^ one used telephone interview^20^, and another principally used telephone interview although a minority used web-based questionnaire.^16^ The PASS study employed a web-based questionnaire issued 14 days after vaccination.^14^ The passive surveillance study collected data from a population-based nominal record.^23^ For one study (evaluating the 2016/17 season in the UK),^19^ the study protocol and an interim analysis were published prior to complete analysis reporting.^24,25^

Methodology slightly varied between studies, likely due to the use of existing infrastructure to collect the information such as EHR system, network or brand-new initiatives with or without ADR cards to collect the information. Nevertheless, those variations may explain the difference reporting rates but unlikely to have altered the nature of the reported events which are mild events reported.

Frequency of AEs and most common local and systemic AEs are presented in Table 1. No safety signals or concerns were identified in the initiatives tabulated.

All studies showed that it is feasible to implement ESS initiatives in the EU and collect near real-time, vaccine-specific reports of AEIs (as well as unsolicited serious adverse events). While this supports the utility of ESS initiatives to the monitoring of adverse events commonly associated with seasonal influenza vaccines, across these studies limited value was demonstrated for capturing serious adverse events or infrequent adverse events. In brief, the studies showed that, in compliance with EMA guidance, ESS is an appropriate method to capture very common (≥1/10), common (≥1/100 to <1/10), and uncommon (≥1/1000 to <1/100) events, but is unlikely to allow detection of rare (≥1/10,000 to <1/1000) or very rare events (<1/10,000). In addition, studies have consistently underscored the significant challenges in capturing data from all age groups as required by the EMA; and in particular in healthy individuals in those age groups not included in the recommended target groups who are thus less likely to receive vaccination.^26^

## Influenza vaccine surveillance initiatives outside the EU

In contrast to the safety surveillance activities mandated in the EU, surveillance initiatives from outside Europe are not conducted by vaccine manufacturers in accordance with regulatory requirements. Instead, they are coordinated by surveillance groups, regulatory bodies or public health representatives. Globally, a variety of different surveillance systems exist; described below are illustrative examples from North America, Oceania, and South America (Table 2).

**Table 2.** Influenza vaccine surveillance outside the EU.

Country	Initiative	Characteristics (including strengths and limitations)	Example of applications
US	VAERS	Health-care providers and vaccine recipients can report any significant health problem following vaccination. VAERS data are monitored to detect new, unusual or rare adverse events and possible safety signals that warrant further evaluation in other studies. Approximately 30,000 VAERS reports are made each year, of which 85−90% are classified as mild (events such as fever, sore arm, and crying or mild irritability) and the remainder are classified as serious, but are rarely proven to be caused by the administered vaccine.^28^ Strengths: its extensive scope and power to detect rare events. Limitations include under- or over-reporting, biased reporting, inconsistency in reporting, inability to assess causality or confirm diagnosis, and inability to calculate the incidence or prevalence of an AEFI.^27^	Annual rapid cycle analysis is used to perform near real-time surveillance of influenza vaccine safety, with weekly monitoring of vaccination records and the occurrence of pre-specified adverse events.^27^ Traditional, retrospective vaccine safety studies are also conducted based on issues identified by the medical literature or reports to VAERS. Following reports in Australia, the CDC and FDA instigated enhanced surveillance for febrile seizure in children for all influenza vaccines; VAERS records were monitored daily for all possible febrile seizure cases in children <5 years, whilst VSD investigators performed weekly rapid cycle analysis for seizures following influenza vaccine.^35^
Canada	CANVAS	The main outcomes of interest are the occurrence of a new health problem or exacerbation of any existing condition severe enough to cause work or school absenteeism, prevent daily activities or require a medical consultation. Over 20,000 individuals participate in active surveillance each year; events occurring at a rate of <1 per 1000 can be detected. Participants receive a web-based survey 8 days after vaccination and any medically attended events are followed up by telephone call.^29^ In 2016, 29,252 adults and parents of immunized children were enrolled in the system, with a survey response rate of 67%.	Evaluation of >20,000 health care workers vaccinated during the 2011 and 2012 influenza seasons, via online questionnaire sent 8 days after vaccination.^36^ Response rate of 69%; participants who reported a severe event were contacted by a nurse for validation purposes. Questionnaire was able to detect events at a frequency of 1 per 1000.^36^
Australia	Adverse Drug Reactions Unit	Notifications are either sent directly or via state health departments. Reports are triaged and reviewed and a causality rating is assigned to each AEFI. Serious, severe, or unexpected events are referred to the Medical Officer. All notifications are reviewed at 6 weekly meetings.	Between January 2000 to September 2002, the reporting rate of AEFI with seasonal influenza vaccine was 3.3 per 100,000 doses in adults 40−64 years of age and 1.4 per 100,000 doses in adults ≥65 years.^30^ Injection site reactions and allergic reactions were most commonly reported.
	SAEFVIC	Enables regional voluntary reporting in the state of Victoria. Established in 2007 and comprises a passive surveillance system coupled with clinical services.^37^	AEFVIC highlighted a possible increase in allergy-related AEFI with 2015 seasonal trivalent influenza vaccines in Australia. An investigation identified a relative risk for allergy-related AEFI of 2.4 per 100,000 vaccine doses in 2015; almost double that seen in 2011−2014 (relative risk of 1.3/100,000), with no difference between vaccine brands.^38^ These findings highlighted the potential for changes in reactogenicity following an update of the influenza vaccine strains, emphasising the importance of continued pharmacovigilance.^38^
	WAVSS	Established in 2011 (built upon the SAEFVIC model) to monitor vaccine safety.^39^ Enables regional voluntary reporting of suspected AEFI from both the public and Health-care providers in the state of Western Australia.	Develops an annual report of brand-specific AEFI in Western Australia.
New Zealand	CARM	Reports are made on a paper form by post and can be submitted by anyone, although notification by a health care professional is preferred.^31^ Community doctors submit 65% of reports, hospital doctors submit 17% and pharmacists submit 2%. The Medicines Adverse Reactions Committee meets four times a year to review published material, all fatal reports and selected reports of significant, unusual or serious reactions reported to CARM.	A review of adverse events with inactivated vaccines reported to CARM between 1990 and 1995 found that more reports of fever were made following Hib (16/100,000) vaccination than following hepatitis B (2/100,000) or influenza (1/100,000) vaccination.^40^
Brazil	ANVISA	Recent enhanced pharmacovigilance initiative to encourage close collaboration between regulators, public health institutes, health care professionals and manufacturers to improve reporting and analysis of AEFI for influenza vaccines.^32,33^ Instructional material to facilitate spontaneous reporting is provided to vaccinees via vaccination clinics, with emphasis on the importance of detecting and reporting AEs to ANVISA.	The objective is to collect AEFI with seasonal influenza vaccines and generate regular reports of adverse events.
Global	WHO survey	Global survey to identify active and passive AEFI surveillance systems for pregnant women and infants. Involves 154 representatives from National Pharmacovigilance Centers and a convenience sample of 31 vaccine safety experts were invited to complete an online survey.^34^	Responses were received from 51 individuals in 47 countries; responding countries represented all WHO regions and included low-, middle- and high-income countries.^34^ Eleven countries had active surveillance systems for detection of serious AEFI in pregnant women or their infants, including six low- or middle-income countries. Passive surveillance systems were identified in 39 countries whose birth cohorts comprise 56% of the global annual birth cohort, including 23 low- or middle-income countries.^34^

AEFI: adverse event following immunization; ANVISA: Agencia Nacional de Vigilancia Sanitaria; CANVAS: Canadian National Vaccine Safety; CARM: Centre for Adverse Reactions Monitoring; SAEFVIC: Surveillance of Adverse Events Following Vaccination in the Community; VAERS: Vaccine Adverse Event Reporting System; WAVSS: Western Australian Vaccine Safety Surveillance

In the US, the Vaccine Adverse Event Reporting System (VAERS) is a national program run by the CDC and the Food and Drug Administration (FDA) that collects reports of adverse events following immunization (AEFI) with all vaccines licensed in the US.^27,28^ Activities include monitoring of known adverse effects for unusual patterns or increases in reporting rate (i.e. number of reports/number of doses), as well as identifying potential associations with new products (e.g. H1N1 vaccine) or in new demographic groups. An alternative system is the Vaccine Safety Datalink (VSD), a collaboration between the CDC and nine integrated health-care organizations.^27^ VSD uses EHRs and other administrative information from health-care organization databases to capture the medical and immunization history from over 9 million people annually (approximately 3% of the US population).^27^

In Canada, the Public Health Agency of Canada (Canadian Institutes of Health Research Influenza Research Network [PCIRN]) was established in 2009 to conduct active safety surveillance of influenza vaccines. In 2014, the Canadian Immunization Research Network which conducts research into immunization programs for all vaccines took over responsibility for influenza, and administers the Canadian National Vaccine Safety (CANVAS) network which monitors AEFI with the seasonal influenza vaccine.^29^

In Australia, the Adverse Drug Reactions Unit has been responsible for passive surveillance of all AEFI since 2000.^30^ AEFI may also be reported at the state level to relevant jurisdictional surveillance systems. In New Zealand, the Centre for Adverse Reactions Monitoring is the national system for all adverse drug reaction reporting.^31^ Recent initiatives have also been instigated in Brazil by the Agencia Nacional de Vigilancia Sanitaria,^32,33^ while a global initiative has been launched by the WHO to better understand the role of maternal immunization (including seasonal influenza) in antenatal care with a global survey to identify national active and passive AEFI surveillance systems across for pregnant women and infants.^34^ Examples of specific applications are presented in Table 2.^27-40^

### Tools in pharmacovigilance and AEFI surveillance

A variety of tools used to improve AEFI surveillance are used in these non-EU initiatives (Table 3). In Australia, the AusVaxSafety surveillance system that uses integrated software to identify and issue automated surveys via text messaging provides active near real-time surveillance capable of rapid data collection on a national level.^41^ The FAST-Mum program has monitored the trivalent influenza vaccine safety in pregnant women in Western Australia since 2012,^42^ while Vaxtracker (also web-based) monitors inactivated influenza vaccine in children in New South Wales.^43^ In Canada, text messaging has been introduced in the CANVAS network as an alternative to conventional telephone interviews.^44^

**Table 3.** Tools used in pharmacovigilance studies of influenza vaccine.

Country	Tool/initiative	Characteristics (including strengths and limitations)	Example of applications
Australia	AusVaxSafety	An automated active near real-time vaccine safety surveillance system. Uses an opt-out monitoring platform integrated with immunization provider software to issue automated surveys to vaccine recipients or caregivers via text messaging. Capable of independently reporting brand-specific data using participant-reported outcomes.	Used to evaluate safety of quadrivalent inactivated influenza vaccine brands for 2017.^41^ Data collected from 73,892 subjects (71.8% of all survey recipients); comparable safety outcomes across brands was demonstrated.^41^
Australia	Text messaging	Text messaging was introduced in the active surveillance FAST-Mum programme in pregnant women in 2013 as an alternative to telephone interview	In one survey, more women responded to text messaging (90%) than to telephone interview (67%).^42^ Timeliness of data collection was improved with text messaging. Despite the higher response rate with text messaging, women surveyed by this method were less likely to report an AEFI than women surveyed by telephone, with the greatest discrepancies being for injection site reactions and unsolicited events.^42^
Australia	Vaxtracker	A web-based active surveillance system in children; automates contact with parents by email or text message to answer 11 symptom questions if the child experienced any kind of reaction after immunization. All serious events are followed up by telephone.	In a study during the 2013 influenza season, of 477 children recruited to use Vaxtracker, 61% of parents completed the survey after the first vaccine dose.^43^ Completion rates were highest when participants provided both email and mobile phone contact details (74%) compared with email (58%) or mobile phone alone (60%). After the first vaccine dose, 8% of respondents reported a local reaction and 3% reported fever. The system allowed rapid analysis of AEFI by health authorities.^43^
Canada	Mobile phone app	Used in the CANVAS network to complete post-vaccination surveys or report AEFI as they occur rather than waiting until the survey is distributed.^39^	Evaluated in a proof of concept study.^44^ A total of 76 people participated, of whom 48 (63%) downloaded the app. Of these 48, 79% completed the day 8 survey, 56% completed the day 30 survey, and 6% completed a joint day 8/30 survey. Eleven participants reported an AEFI, including one spontaneous report that was also reported using the day 8 survey.^44^ Twenty-one participants completed a usability survey, of whom 86% agreed or strongly agreed that they preferred an app over a web-based system.
US	Text-mining	Used in VAERS for automated classification of reports	In one study, medical officers evaluated 6034 VAERS reports for H1N1 vaccine to determine whether the reports met the Brighton Collaboration case definition for anaphylaxis Text-mining techniques extracted three feature sets, i.e. important key words, low-level patterns and high-level patterns. The authors concluded that this approach could be applied effectively to VAERS data, potentially reducing staff workload and providing more timely information.^45^
Multinational	Time-to-onset methodology	Used in a GSK database (spontaneous reports of EFI with rotavirus and influenza vaccines). Adverse events were identified as safety signals if their time-to-event distribution was significantly different from the distribution of other events with the same vaccine or from the distribution of the same event for other vaccines.^46^ The product label was used as a proxy to evaluate a realistic threshold for safety signals.	For the influenza vaccine, 36 safety signals were identified (based on Medical Dictionary for Regulatory Activities preferred terms), of which 11 appeared in the product label (i.e. a true positive signal). This compared with four preferred terms identified using disproportionality analysis, none of which were in the product label. It appeared that the time-to-event method had a higher sensitivity compared with the standard disproportionality method (14.5% versus 0%), but slightly lower specificity (98.0% versus 99.7%).^46^

AEFI: adverse event following immunization; CANVAS: Canadian National Vaccine Safety; FAST-Mum: Follow-up and Active Surveillance of Trivalent influenza vaccine in Mums; VAERS: Vaccine Adverse Event Reporting System

It is difficult in the context of current safety surveillance systems to process data in a timely manner, particularly during events such as an influenza pandemic, because the manual review of events is time-consuming and resource-intensive. In the US, a recent initiative by VAERS has employed a text-mining approach to provide automated text classification of AEFI reports for H1N1 vaccine to offer a more efficient way to identify the most relevant information.^45^

In routine pharmacovigilance, disproportionality analyses are the most common methods of analyzing spontaneous reports. However, some adverse events known to have a causal relationship with vaccination typically occur within a particular time window post-vaccination (e.g. febrile convulsion, intussusception) and disproportionality analyses do not take the time-to-onset of the adverse event into account. Use of alternative time-to-onset methodology may provide an alternative approach in identifying adverse events, as recently reported in a proof-of-concept study evaluating AEFIs and influenza vaccination.^46^

## Lessons learned and future perspectives

**Figure 1.**
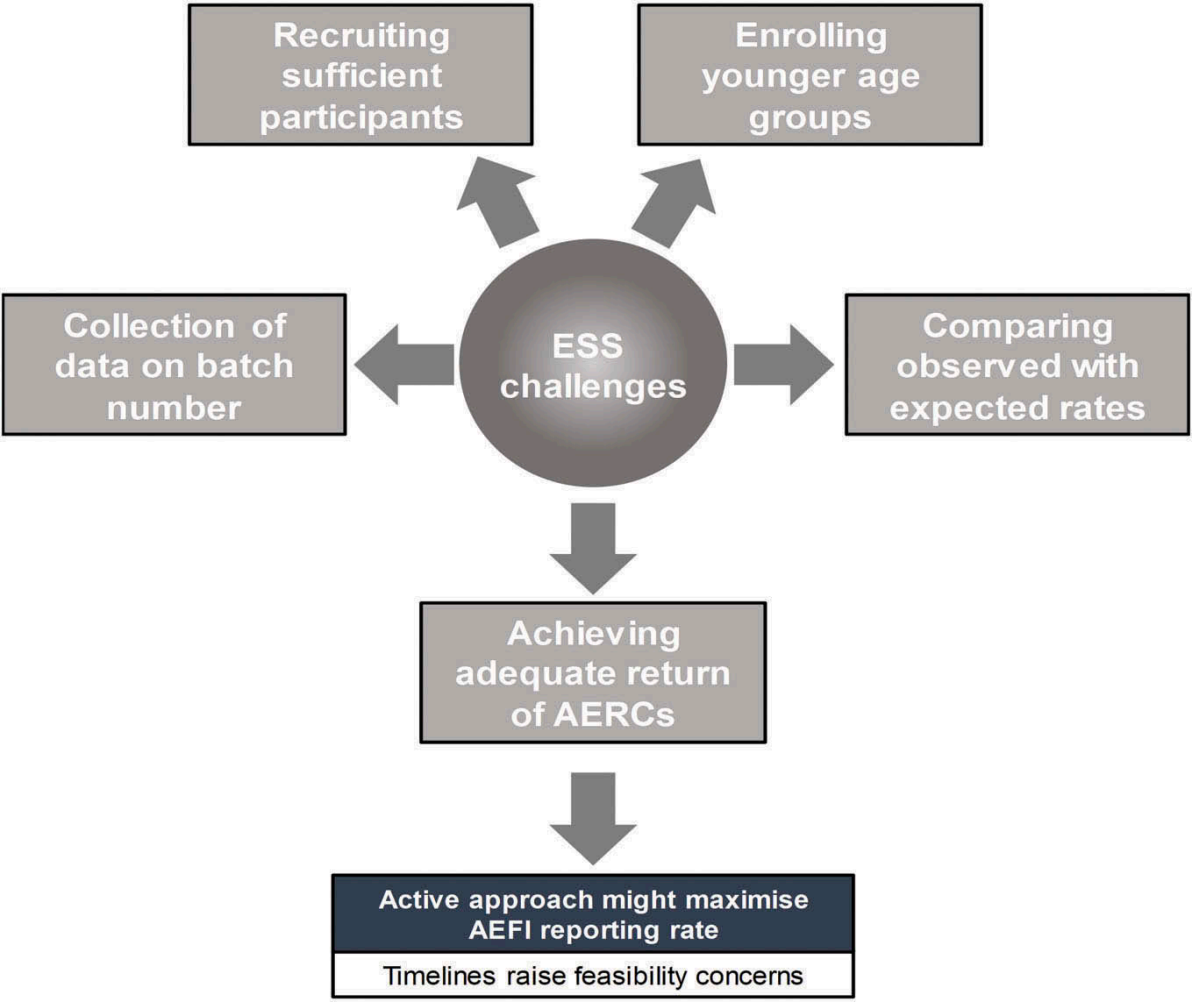
Challenges in implementing ESS in the EU. AEFI: adverse event following immunization; AERC: adverse event reporting card; ESS: enhanced safety surveillance; EU: European Union

Described here are several initiatives to monitor AEFI, with details of their scope and the tools used to contribute to a better understanding of the safety profile of seasonal influenza vaccines. In Europe, the ESS studies described identified a number of challenges and limitations in implementing such initiatives annually (Figure 1). Recruiting sufficient participant numbers is particularly challenging. A sample size of 1000 participants would be expected to allow detection of the most common events, but less likely to capture the rarer ones. The probability of observing at least one AEI and the associated level of precision for a range of sample sizes and other factors has been discussed in depth elsewhere. A sample size calculation reported in a published study showed for instant the probability of observing at least one AEI during the study period and he the associated relative standard error (RSE) for a range of scenarios in terms of cohort size, vaccine coverage, and expected probability of AEI. With an overall sample size of a minimum of about 50,000 subjects medically followed by the enrolled General Practices, a follow-up period of 14 weeks, a vaccine coverage of 5%, 10%, or 20% and an expected probability of AEI varying from 0.01% to 20%, the corresponding probability to observe at least one event varies from 2% to 100%, and the associated RSE varies from 2% to 200% depending on the scenario.^24^ EMA guidance requires surveillance to be conducted in settings that allow all age groups covered by the product license to be enrolled. However, enrolment of younger age groups is especially difficult, in particular in countries which have a preferential recommendation for only one type of vaccine for this age group (e.g. live attenuated intranasal vaccine is used for children in the UK).

Reliable and comprehensive collection of batch numbers can also present difficulties depending on the source of detailed vaccination records. One UK study reported data integrity issues or suboptimal communication from pharmacists to general practitioners; including infrequent reporting of vaccine brand or batch number, and poor key coding data entry into EHR systems, which can lead to dissimilarity in data reconciliation.^47^ Lack of essential information such as vaccine brand or batch numbers is likely to preclude or limit reliable monitoring of vaccine safety or effectiveness studies. As part of ‘Good Pharmacovigilance Practices’ the EMA recommends that the product name and batch number are recorded throughout the supply chain from manufacturer release to administration, and emphasizes that traceability should be fully implemented despite variation across products and between countries in health-care settings and infrastructure.^48^ With this in mind, national health authorities are encouraged to work towards better identification, awareness, integration, and automation of prescription information. The European Commission has adopted a new regulation (EU 2016/161, supplement to Directive 2001/83/EC) imposing a requirement for all pharmaceutical products to have an authentication process, based on serial numbers on individual pharma packages, with a deadline of February 2019 for member states (with some exceptions).^49^ Although not an initial objective of the regulation, this initiative is expected to bring added value and consistency to the conduct of vaccine safety monitoring studies.

In the absence of pre-defined adverse event baseline rates at the start of an ESS study, the EMA recommends comparing rates obtained during the course of the surveillance period with the expected rates contained in the Summary of Product Characteristics (SmPC) and/or the data obtained in the previous year of ESS using an identical method for surveillance.^4^ As it is not always possible to conduct an ESS study in the same country every year, this limits the possibility of using ESS data from the previous year as a comparator, and rates listed in the SmPC may be a more reliable benchmark.

Under-reporting of adverse events is a common challenge of routine pharmacovigilance.^50^ Use of EPS employing an AERC helps to reduce this problem, but the return rate of AERCs remains suboptimal, and it is likely that some events, particularly relatively minor events, remain under-reported.^51^ EPS could benefit from a more active approach to overcome suboptimal reporting, however this poses logistical constraints and raises questions as to the sustainability of such approach. Consequently, although an active approach might maximize the reporting of AEIs, these timelines raise feasibility concerns, especially considering the requirement to implement this initiative on a yearly basis and EPS remains the preferred option. In 2016, Vaccines Europe and Vaccines Working Party were consulted to better understand how the guidance could best be applied. The European market is challenging to forecast well in advance of the season, and the countries able to host such study are unlikely to be identified prior to the first quarter of each year.

The annual assessment of the safety of seasonal influenza vaccines presents particular challenges. Different stakeholders such as regulators, manufacturers, and public health bodies have attempted to address these challenges in varying ways. However, several existing tools or initiatives could be complementary. For instance, building on enhanced pharmacovigilance, as described elsewhere,^52-54^ could potentially provide a more sustainable, informed and continuous assessment using automated signal detection and evaluation tools, and thus deserves to be investigated further.

Attempting to harmonize the standards of safety monitoring across regions and countries and creating synergies is an important element of influenza surveillance. Such harmonization would, for instance, allow one hemisphere to build on findings from the other, assuming comparable immunization populations and that the strain composition of the vaccine remains unchanged between hemispheres, as briefly described in the interim EMA guidance.^4^ Furthermore, enhancing pharmacovigilance inspiring from the Global Vaccine Safety initiative^54^ or creating synergies between routine pharmacovigilance and ESS initiatives could potentially allow a more sustainable and comprehensive assessment, as well as larger scale evaluations. However, such initiatives would require a closer collaboration between relevant stakeholders, including public health institutes or surveillance initiatives, vaccine manufacturers and regulators, ideally at national, regional and global levels. The ultimate purpose would be to find the best model to efficiently monitor the safety of seasonal influenza vaccines, leading to an appropriate and timely response if a safety concern arises.

In conclusion, despite the plethora of existing tools and initiatives, it is acknowledged that ESS may bring useful elements to the evaluation of near real-time safety of newly formulated seasonal influenza vaccines and confirmation of the safety profile as reported in the SmPC. However, it is probably premature to determine whether or not this initiative brings true added value compared with the previous small-scale safety and immunogenicity clinical trials or whether other pharmacovigilance initiatives could not also provide meaningful elements to address similar objectives. Close dialogue between the key actors in the influenza field is likely to further inform the medical and regulatory processes concerning vaccine safety.

## Declaration Statements

**Trademark section**

Fluvax is a trademark of CSL Biotherapies. Fluad is a trademark of Seqirus. Intanza, Vaxigrip, and Vaxigrip Tetra are trademarks of Sanofi Pasteur. Fluarix Tetra and Alpharix Tetra are trademarks of the GSK group of companies. Fluenz Tetra is a trademark of AstraZeneca.
